# Supplementary figures and images for: Nocturnal substrate association of four coral reef fish groups (parrotfishes, surgeonfishes, groupers and butterflyfishes) in relation to substrate architectural characteristics
Source: PeerJ. 2024 Jul 19;12:e17772. doi: 10.7717/peerj.17772 (PMC11262305; doi:10.7717/peerj.17772)

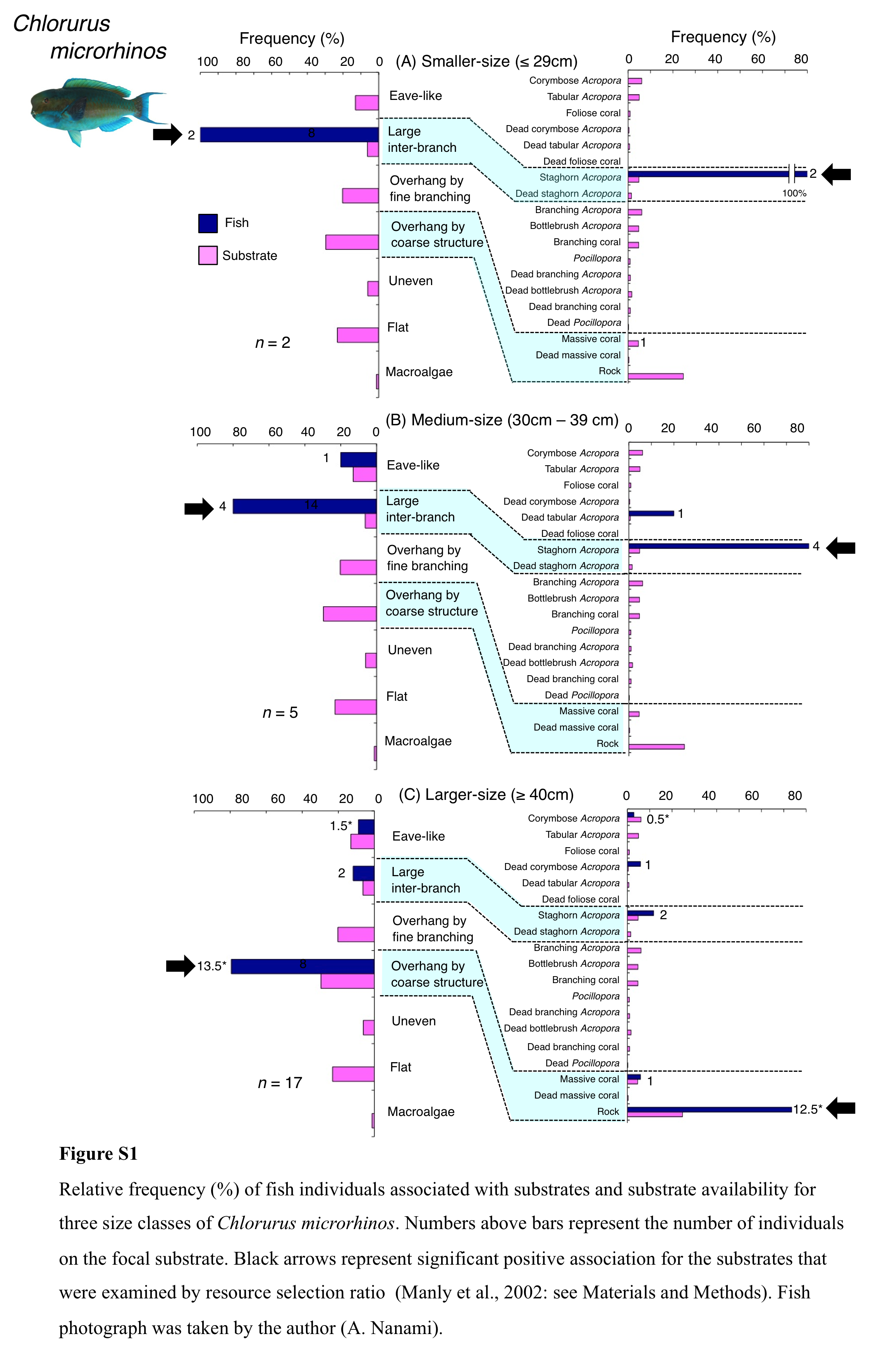

Supplement: Supplemental Information 1 — Numbers above bars represent the number of individuals on the focal substrate. Black arrows represent significant positive association for the substrates that were examined by resource selection ratio (Manly et al., 2002: see Materials and Methods). Fish photograph was taken by the author (A. Nanami). [file peerj-12-17772-s001.png]

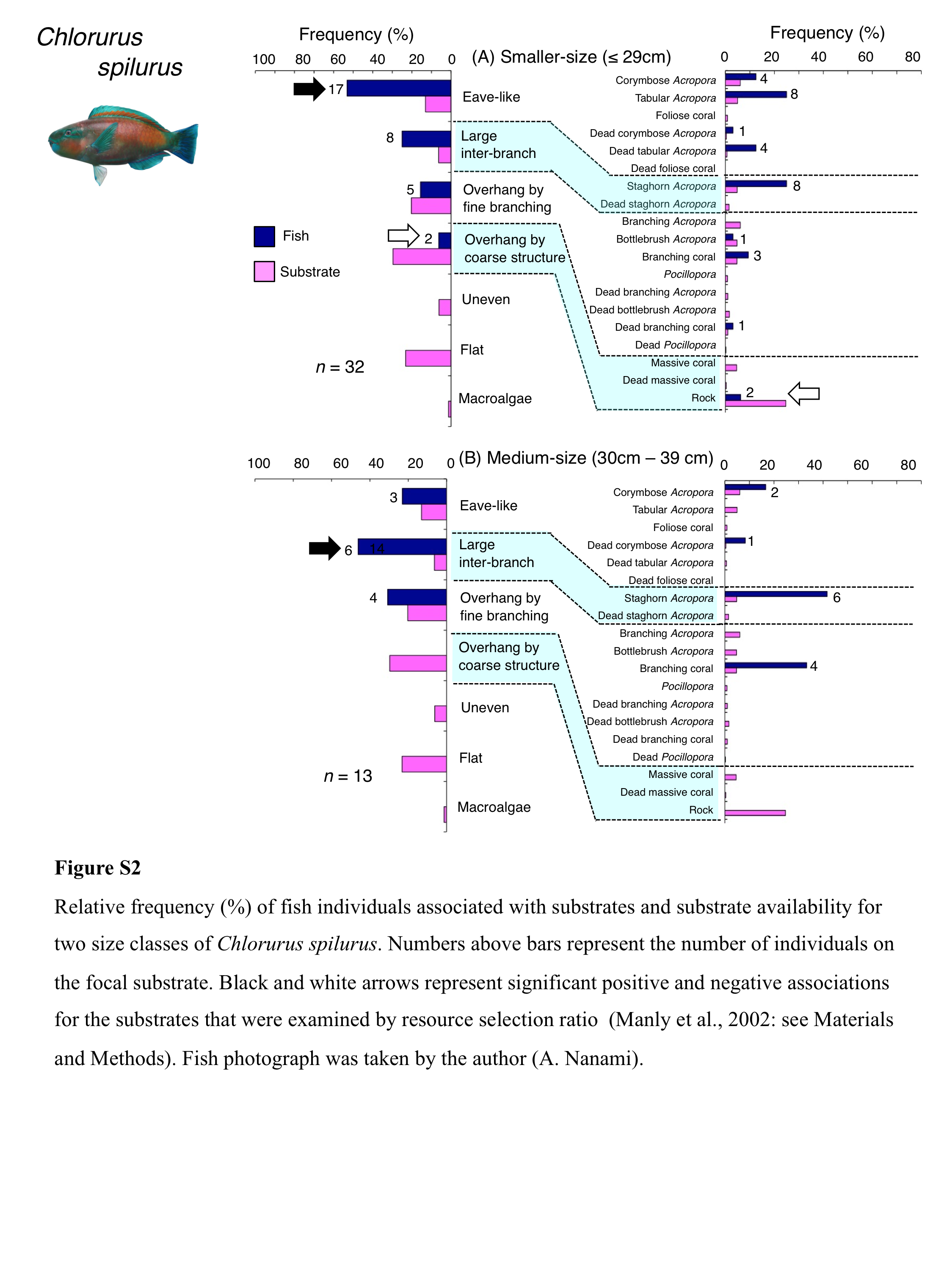

Supplement: Supplemental Information 2 — Numbers above bars represent the number of individuals on the focal substrate. Black and white arrows represent significant positive and negative associations for the substrates that were examined by resource selection ratio (Manly et al., 2002: see Materials and Methods). Fish photograph was taken by the author (A. Nanami). [file peerj-12-17772-s002.png]

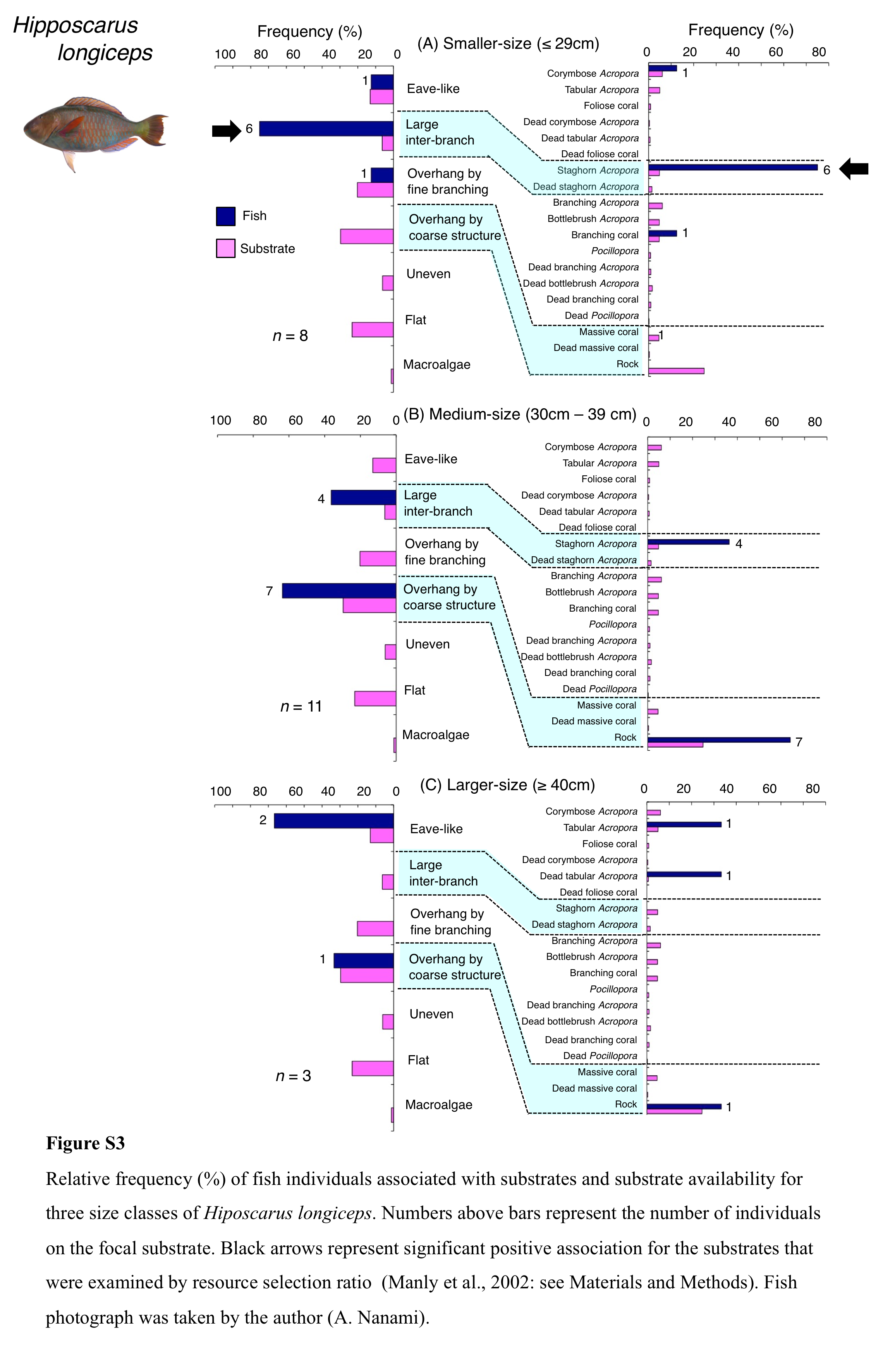

Supplement: Supplemental Information 3 — Numbers above bars represent the number of individuals on the focal substrate. Black arrows represent significant positive association for the substrates that were examined by resource selection ratio (Manly et al., 2002: see Materials and Methods). Fish photograph was taken by the author (A. Nanami). [file peerj-12-17772-s003.png]

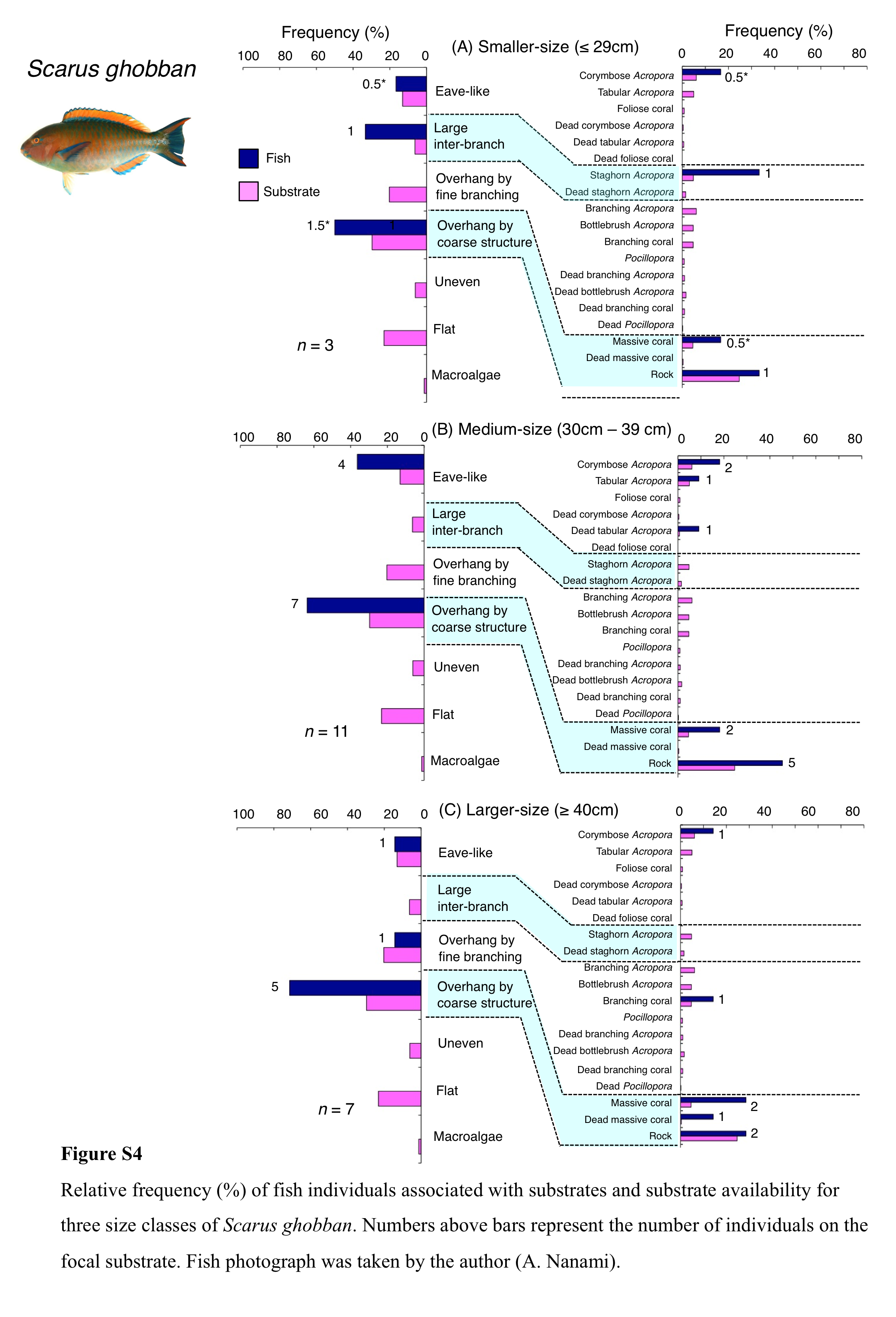

Supplement: Supplemental Information 4 — Numbers above bars represent the number of individuals on the focal substrate. Fish photograph was taken by the author (A. Nanami). [file peerj-12-17772-s004.png]

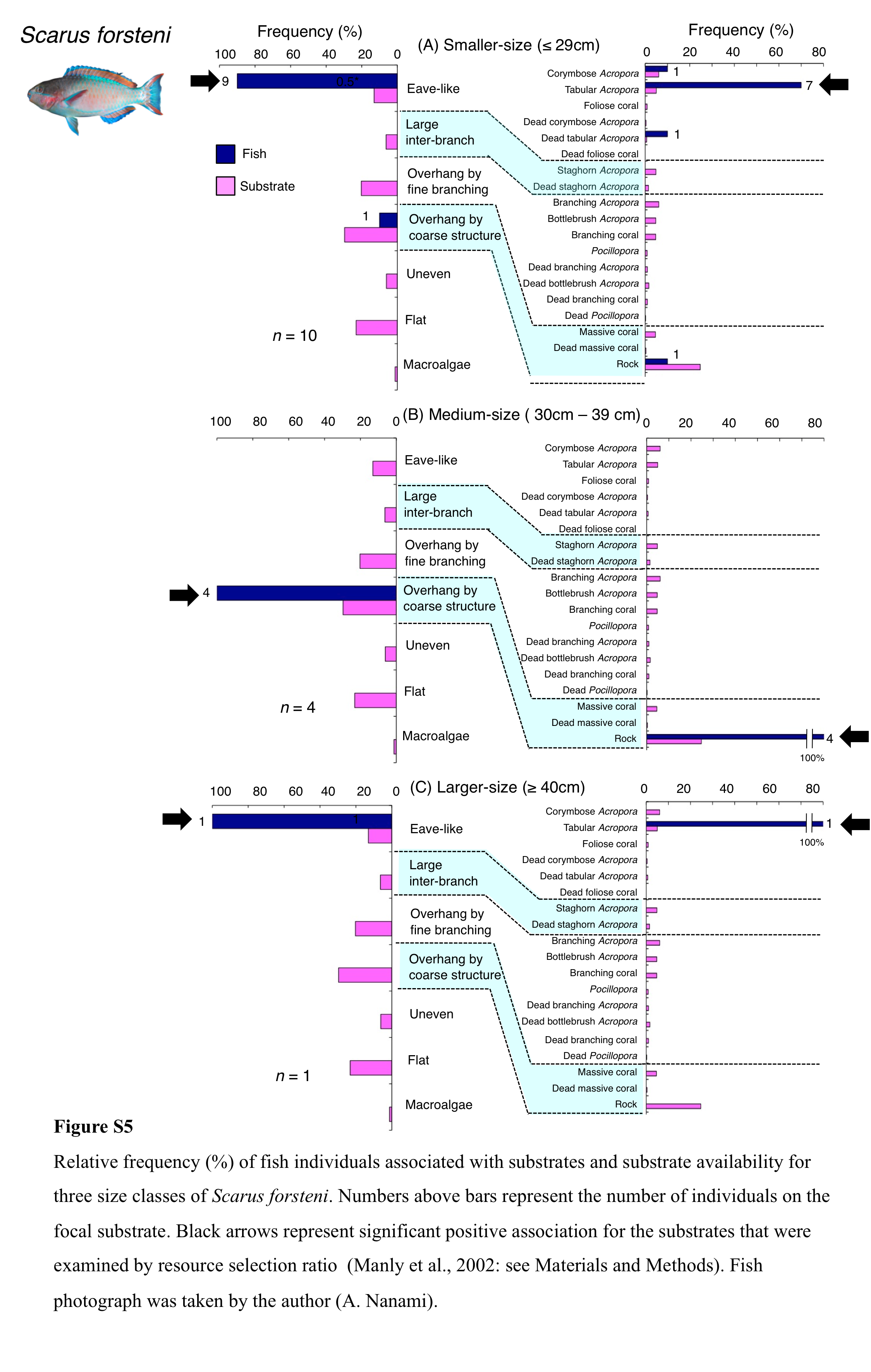

Supplement: Supplemental Information 5 — Numbers above bars represent the number of individuals on the focal substrate. Black arrows represent significant positive association for the substrates that were examined by resource selection ratio (Manly et al., 2002: see Materials and Methods). Fish photograph was taken by the author (A. Nanami). [file peerj-12-17772-s005.png]

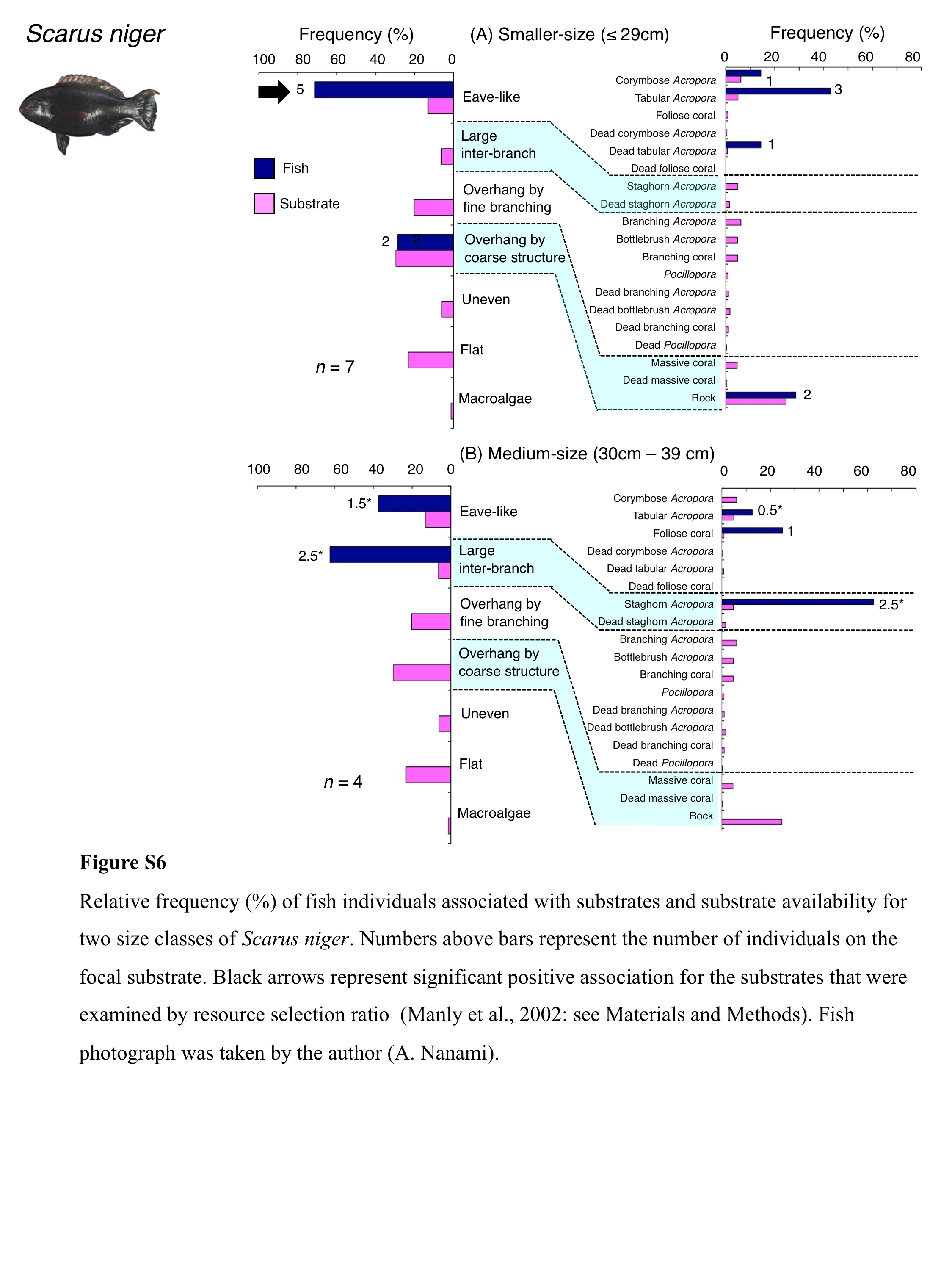

Supplement: Supplemental Information 6 — Numbers above bars represent the number of individuals on the focal substrate. Black arrows represent significant positive association for the substrates that were examined by resource selection ratio (Manly et al., 2002: see Materials and Methods). Fish photograph was taken by the author (A. Nanami). [file peerj-12-17772-s006.png]

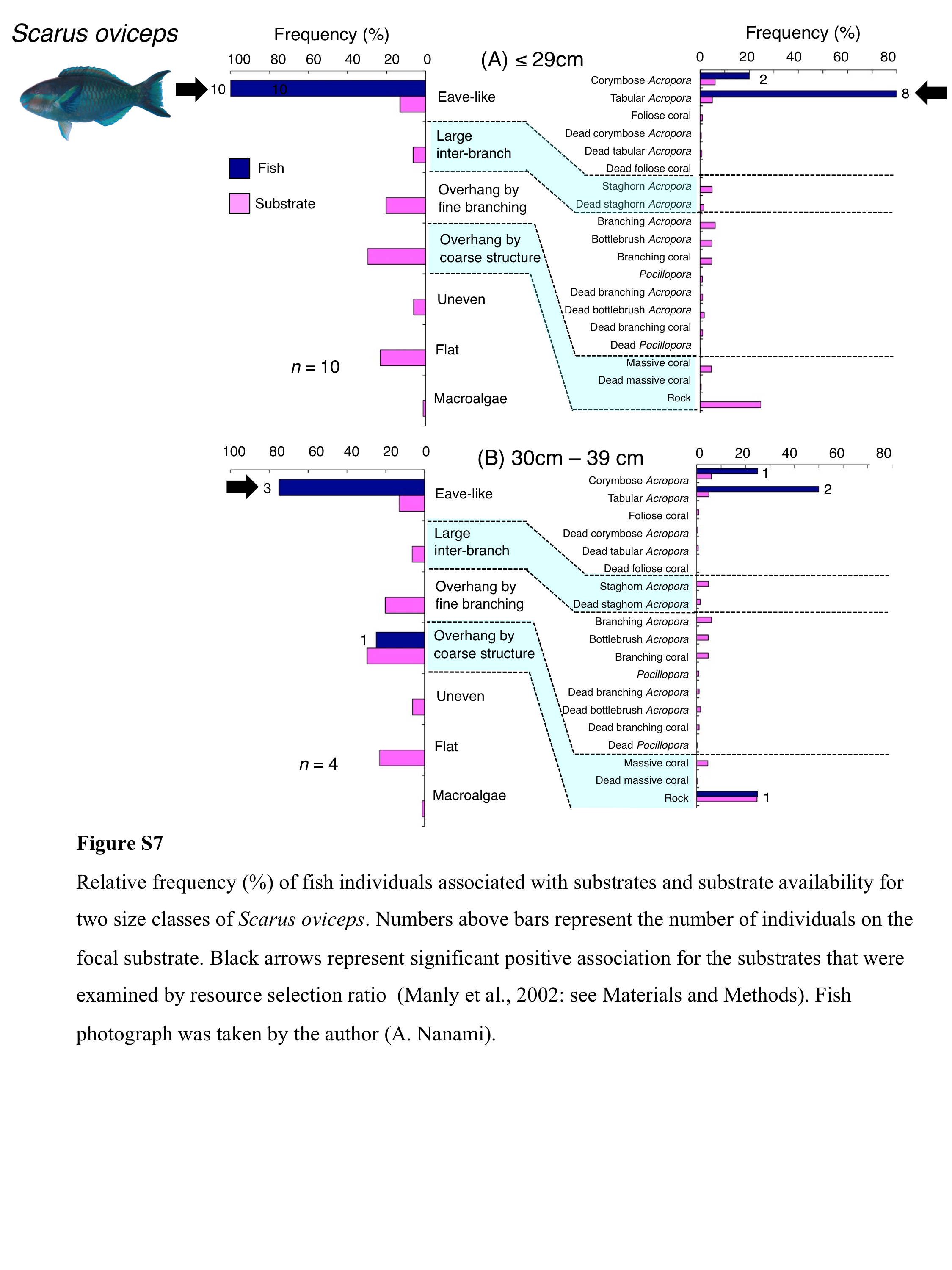

Supplement: Supplemental Information 7 — Numbers above bars represent the number of individuals on the focal substrate. Black arrows represent significant positive association for the substrates that were examined by resource selection ratio (Manly et al., 2002: see Materials and Methods). Fish photograph was taken by the author (A. Nanami). [file peerj-12-17772-s007.png]

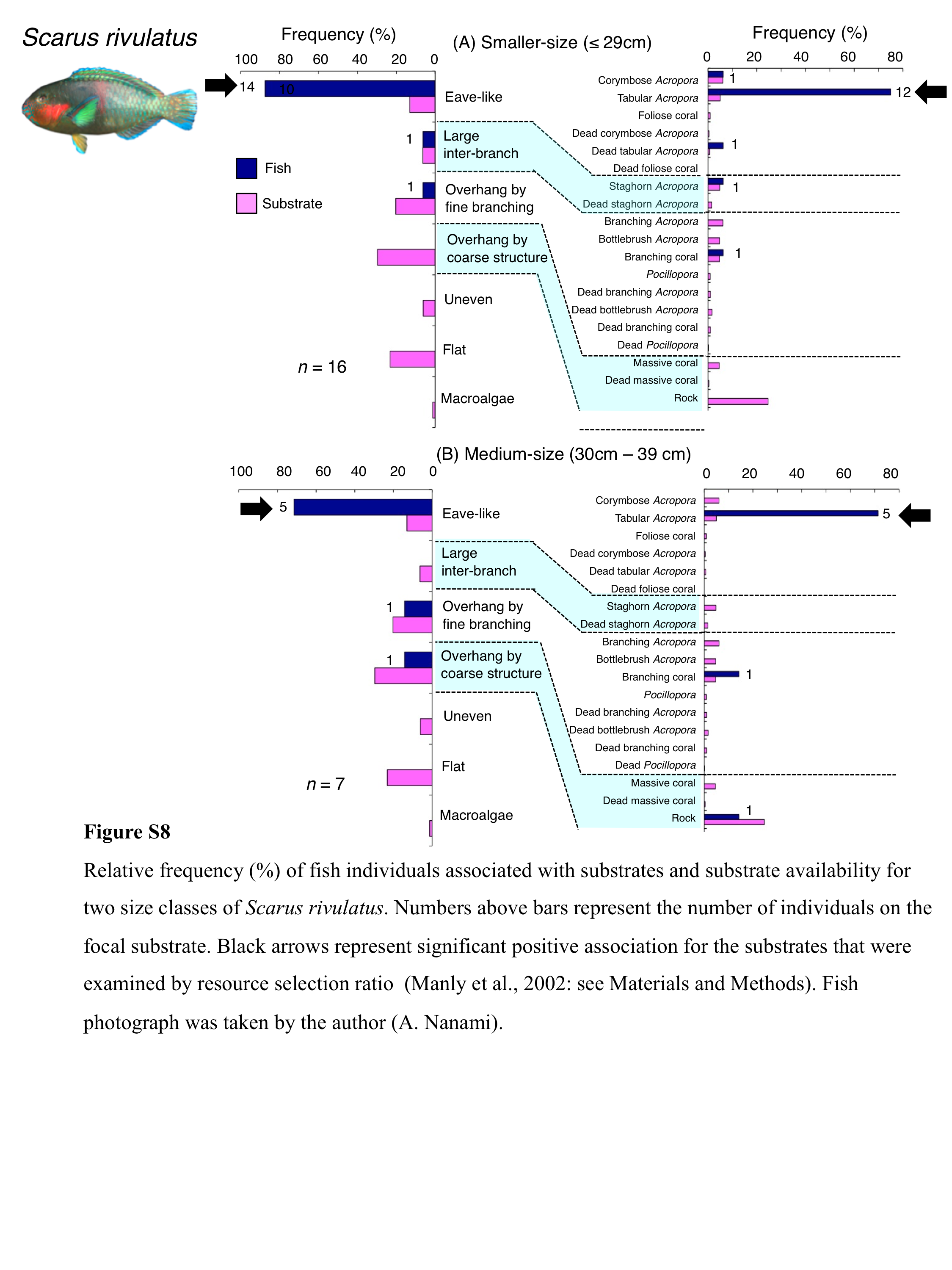

Supplement: Supplemental Information 8 — Numbers above bars represent the number of individuals on the focal substrate. Black arrows represent significant positive association for the substrates that were examined by resource selection ratio (Manly et al., 2002: see Materials and Methods). Fish photograph was taken by the author (A. Nanami). [file peerj-12-17772-s008.png]

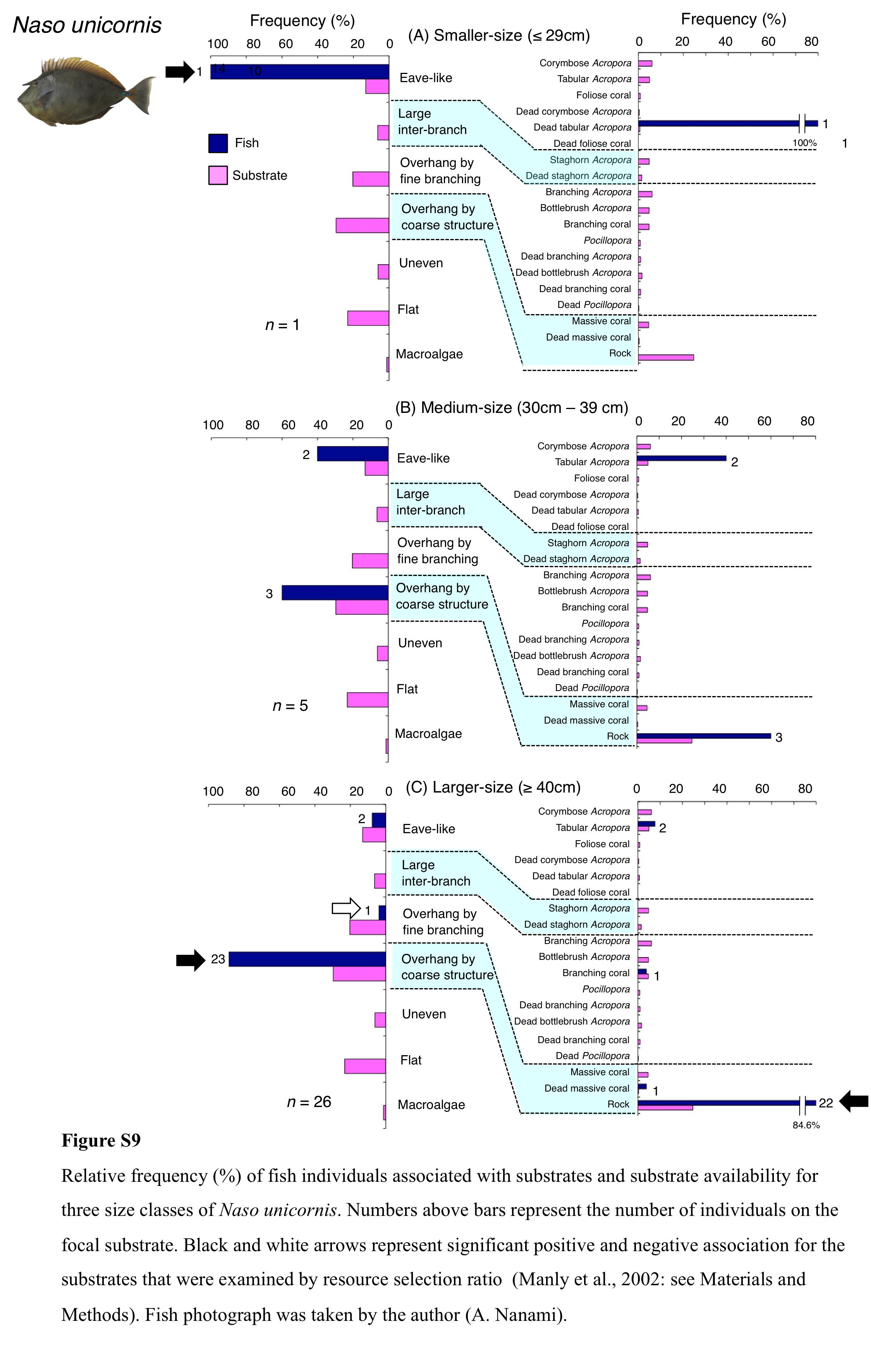

Supplement: Supplemental Information 9 — Numbers above bars represent the number of individuals on the focal substrate. Black and white arrows represent significant positive and negative association for the substrates that were examined by resource selection ratio (Manly et al., 2002: see Materials and Methods). Fish photograph was taken by the author (A. Nanami). [file peerj-12-17772-s009.png]

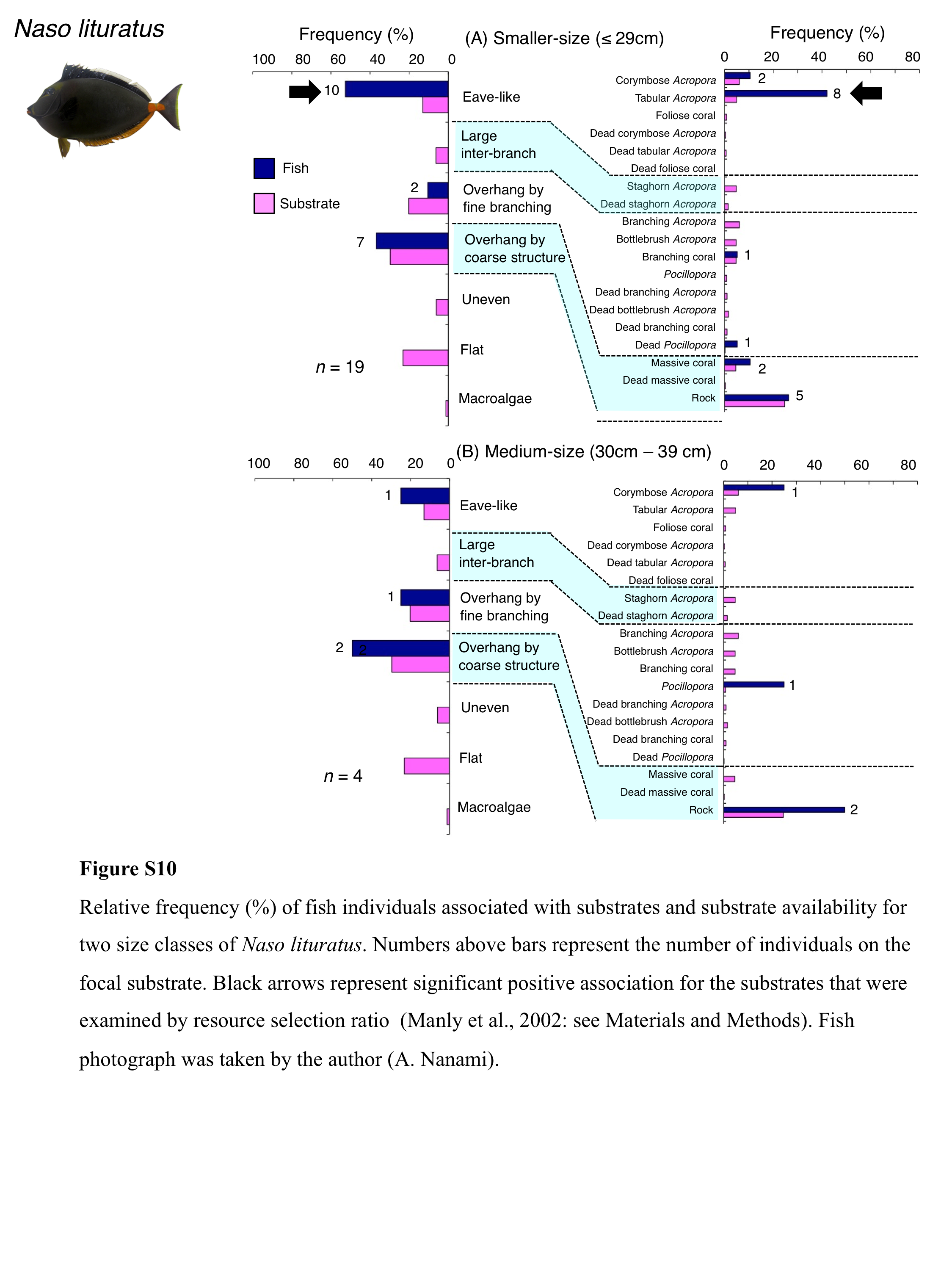

Supplement: Supplemental Information 10 — Numbers above bars represent the number of individuals on the focal substrate. Black arrows represent significant positive association for the substrates that were examined by resource selection ratio (Manly et al., 2002: see Materials and Methods). Fish photograph was taken by the author (A. Nanami). [file peerj-12-17772-s010.png]

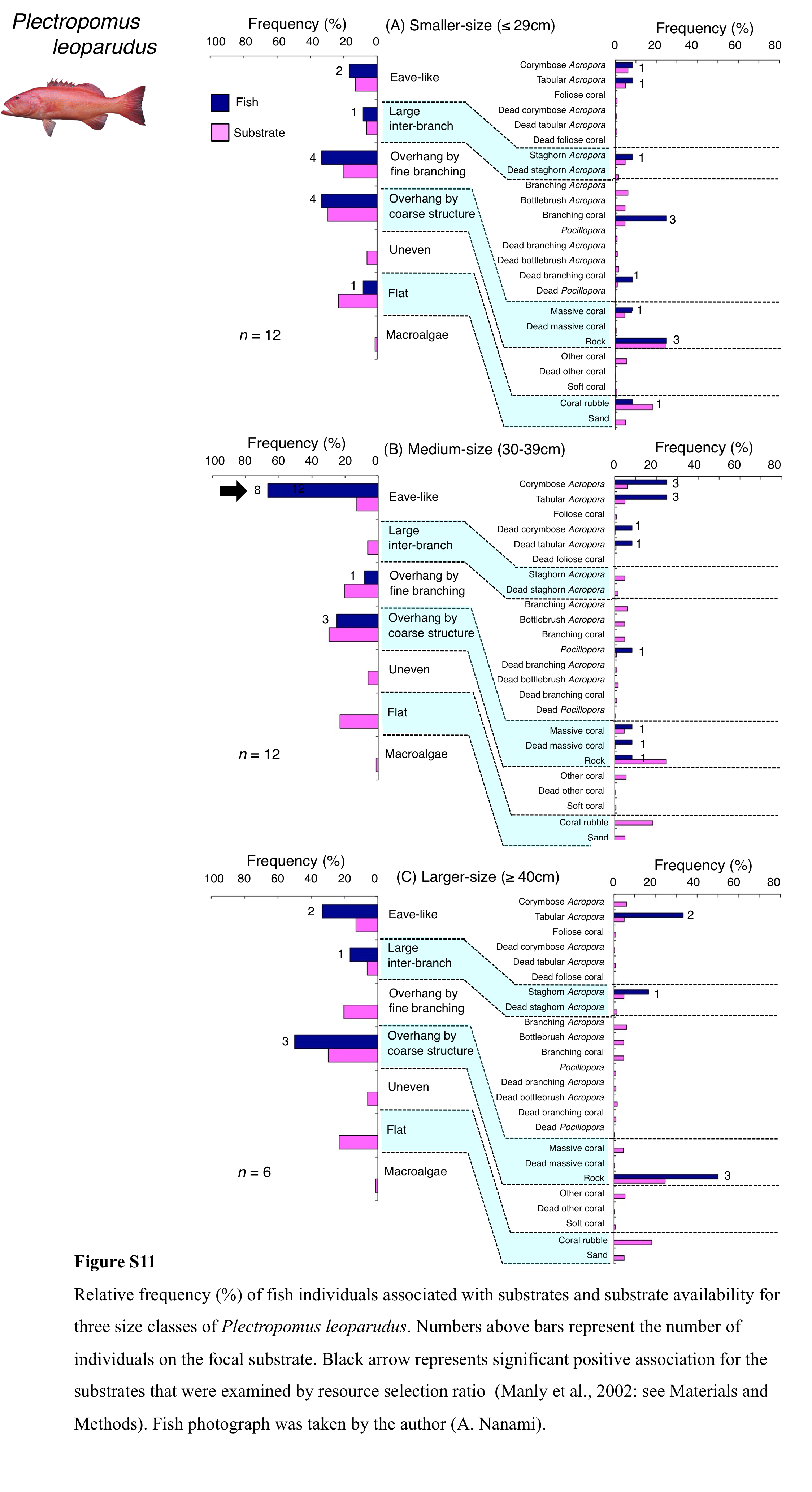

Supplement: Supplemental Information 11 — Numbers above bars represent the number of individuals on the focal substrate. Black arrow represents significant positive association for the substrates that were examined by resource selection ratio (Manly et al., 2002: see Materials and Methods). Fish photograph was taken by the author (A. Nanami). [file peerj-12-17772-s011.png]

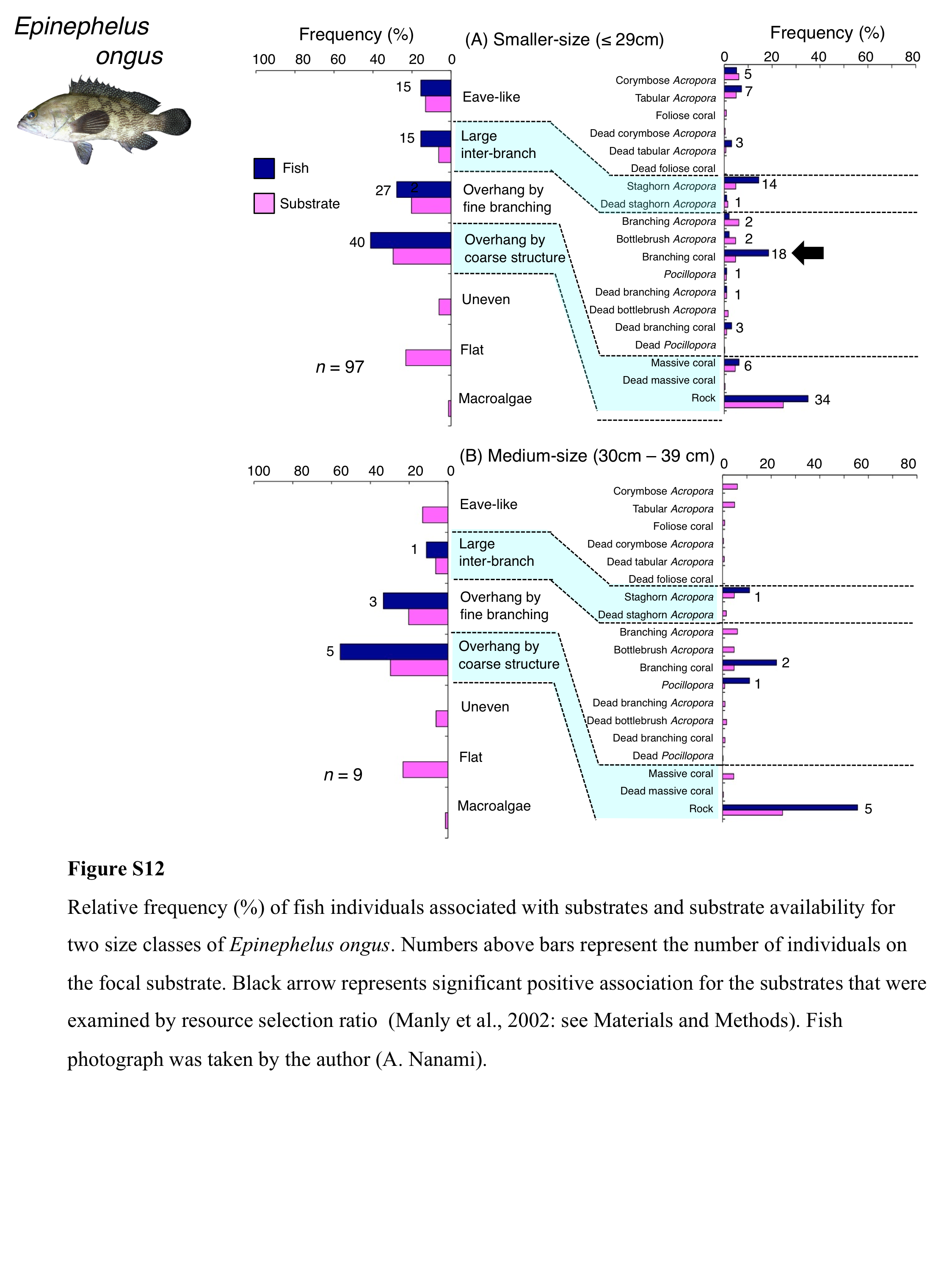

Supplement: Supplemental Information 12 — Numbers above bars represent the number of individuals on the focal substrate. Black arrow represents significant positive association for the substrates that were examined by resource selection ratio (Manly et al., 2002: see Materials and Methods). Fish photograph was taken by the author (A. Nanami). [file peerj-12-17772-s012.png]

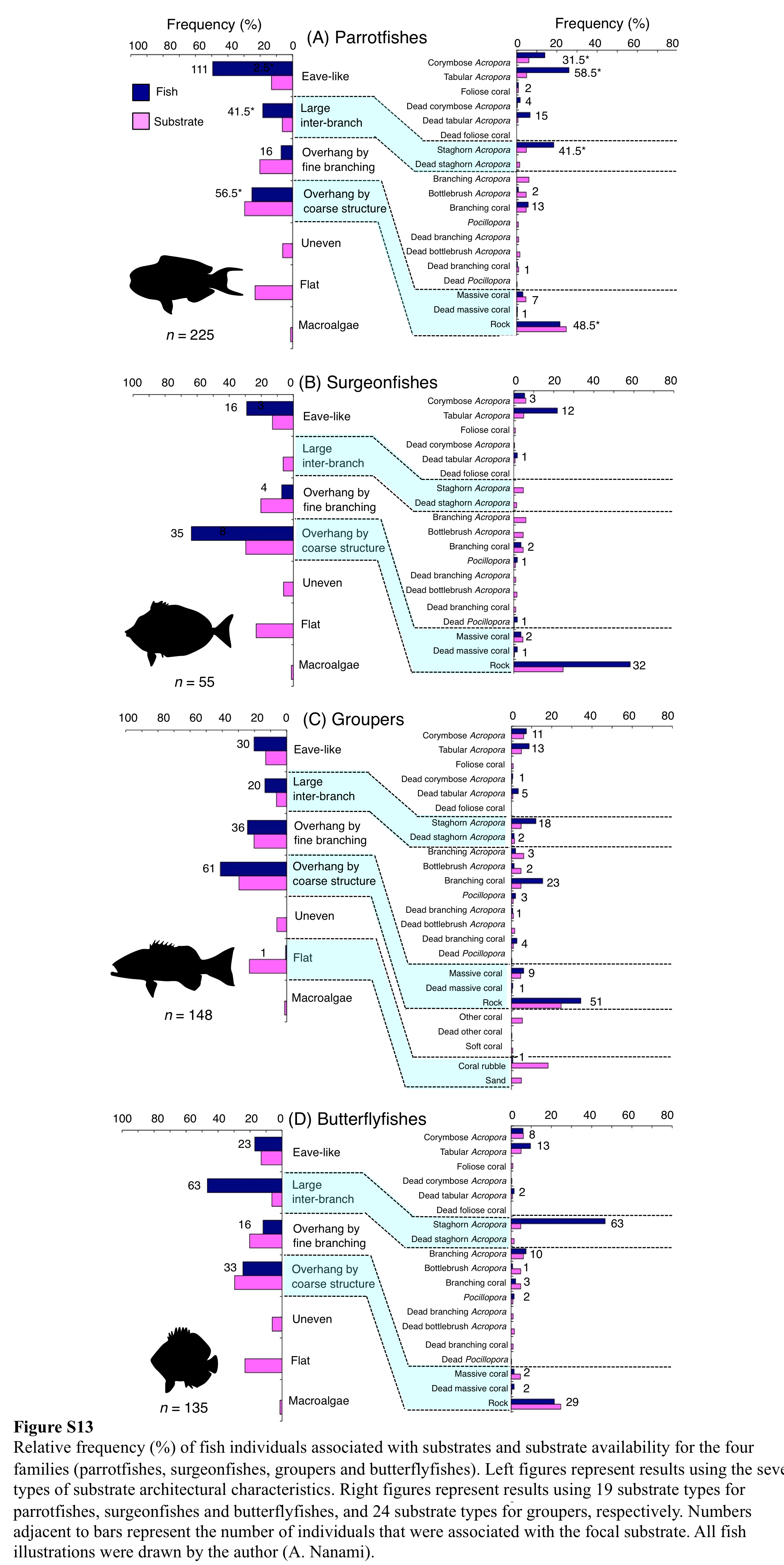

Supplement: Supplemental Information 13 — Left figures represent results using the seven types of substrate architectural characteristics. Right figures represent results using 19 substrate types for parrotfishes, surgeonfishes and butterflyfishes, and 24 substrate types for groupers, respectively. Numbers adjacent to bars represent the number of individuals that were associated with the focal substrate. All fish illustrations were drawn by the author (A. Nanami). [file peerj-12-17772-s013.png]

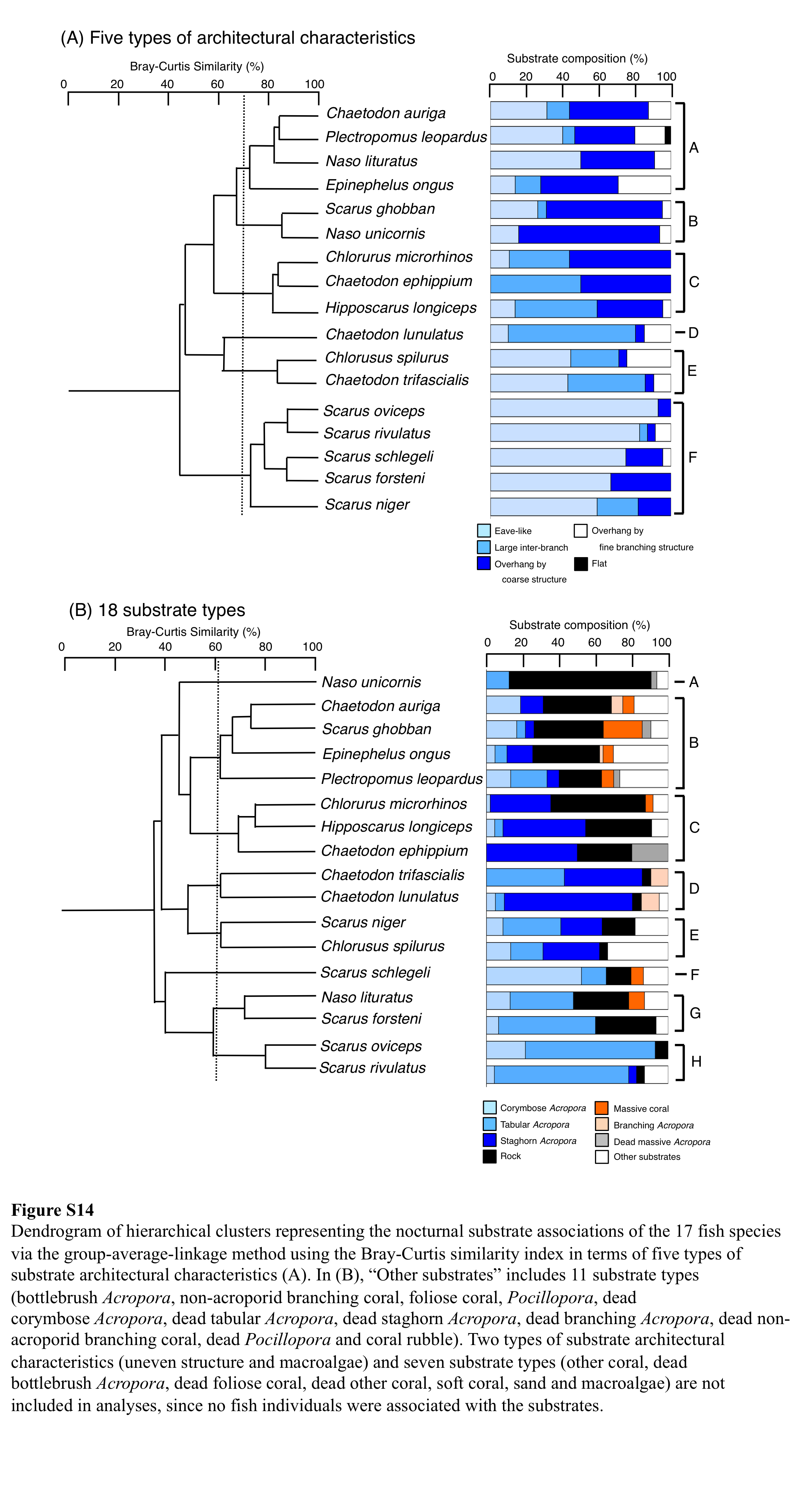

Supplement: Supplemental Information 14 — In (B), “Other substrates” includes 11 substrate types (bottlebrush Acropora, non-acroporid branching coral, foliose coral, Pocillopora, dead corymbose Acropora, dead tabular Acropora, dead staghorn Acropora, dead branching Acropora, dead non-acroporid branching coral, dead Pocillopora and coral rubble). Two types of substrate architectural characteristics (uneven structure and macroalgae) and seven substrate types (other coral, dead bottlebrush Acropora, dead foliose coral, dead other coral, soft coral, sand and macroalgae) are not included in analyses, since no fish individuals were associated with the substrates. For details about data, see ” Fig. S14 raw data.xls.” [file peerj-12-17772-s014.png]

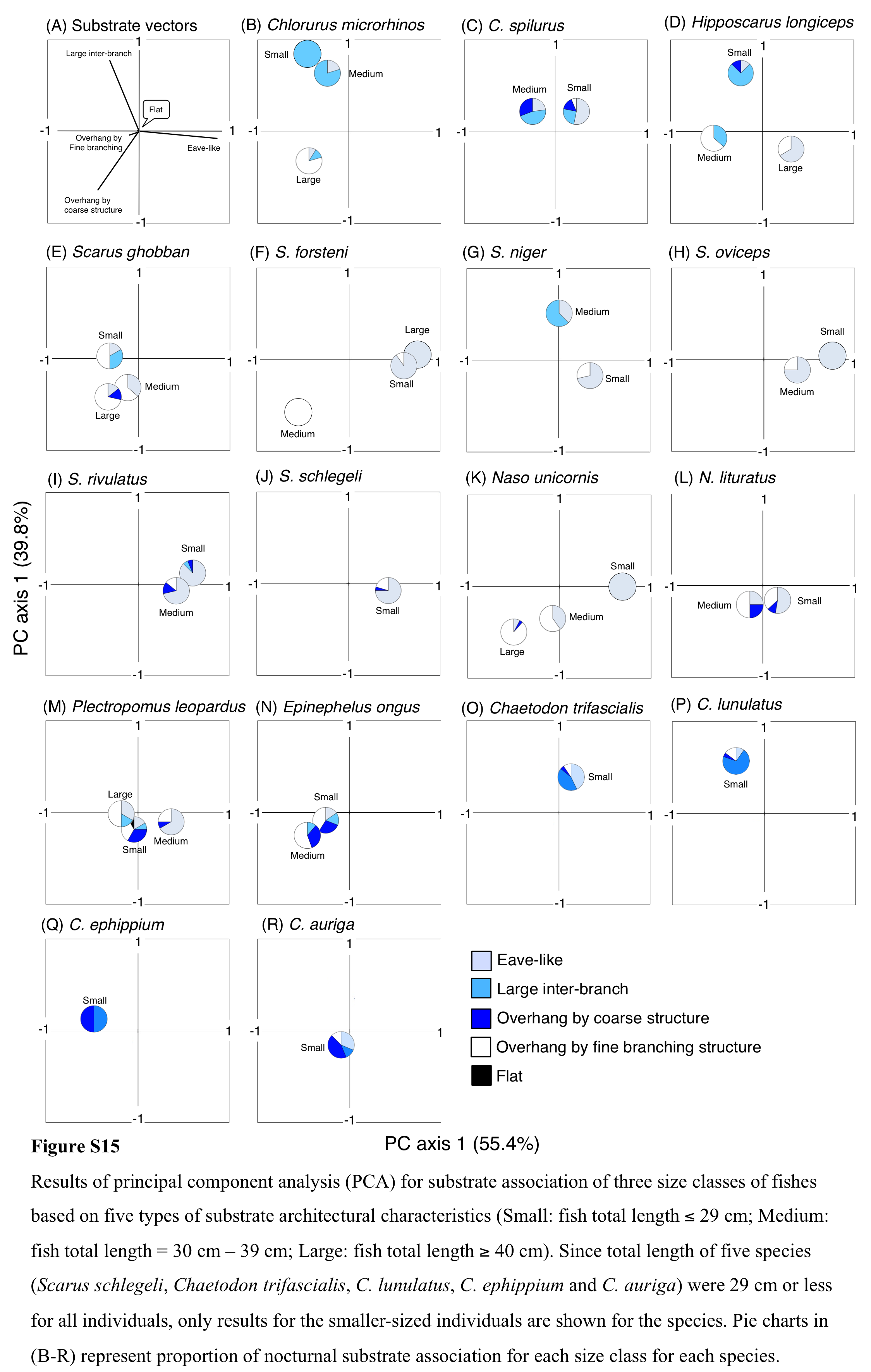

Supplement: Supplemental Information 15 — Since total length of five species (Scarus schlegeli, Chaetodon trifascialis, C. lunulatus, C. ephippium and C. auriga) were 29 cm or less for all individuals, only results for the smaller-sized individuals are shown for the species. Pie charts in (B-R) represent proportion of nocturnal substrate association for each size class for each species. [file peerj-12-17772-s015.png]

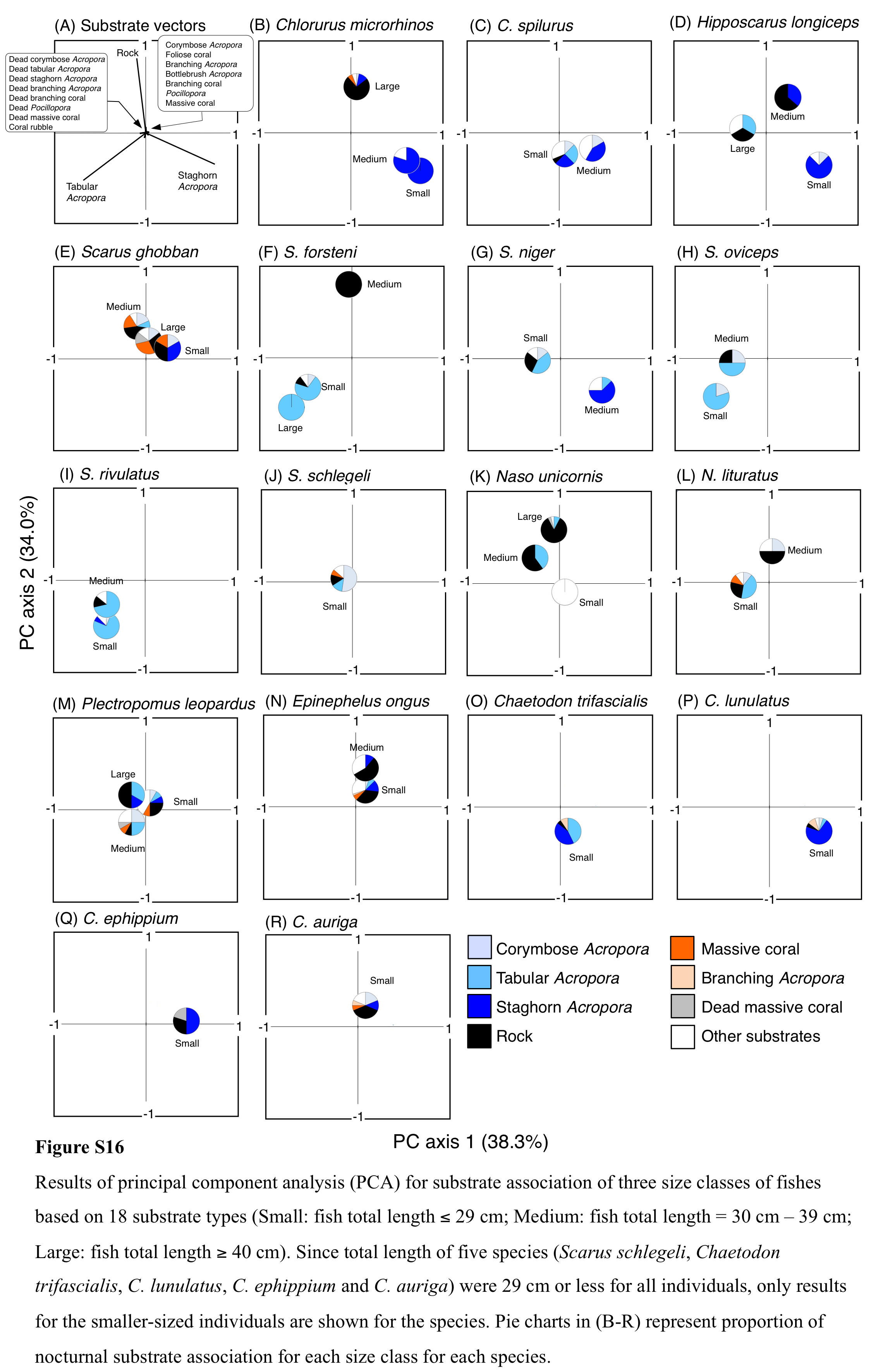

Supplement: Supplemental Information 16 — Since total length of five species (Scarus schlegeli, Chaetodon trifascialis, C. lunulatus, C. ephippium and C. auriga) were 29 cm or less for all individuals, only results for the smaller-sized individuals are shown for the species. Pie charts in (B-R) represent proportion of nocturnal substrate association for each size class for each species. [file peerj-12-17772-s016.png]
