# Supplementary material for: Nocturnal substrate association of four coral reef fish groups (parrotfishes, surgeonfishes, groupers and butterflyfishes) in relation to substrate architectural characteristics
Source: PeerJ. 2024 Jul 19;12:e17772. doi: 10.7717/peerj.17772 (PMC11262305; doi:10.7717/peerj.17772)
Supplement: Supplemental Information 17 — Significant positive associations are shown as bold characters. N.S.: non significant associations. -: no fishes were found on the substrates. [file peerj-12-17772-s017.docx]

|  |  |  |  |  |  |  |  |  |  |
| --- | --- | --- | --- | --- | --- | --- | --- | --- | --- |
| Substrate  architectural characteristics | *Chlorurus microrhinos* | *Chlorurus spilurus* | *Hipposcarus longiceps* | *Scarus ghobban* | *Scarus forsteni* | *Scarus niger* | *Scarus oviceps* | *Scarus rivulatus* | *Scarus schlegeli* |
| Eave-like | 0.100 | **0.360** | 0.108 | 0.819 | **0.389** | **0.519** | **0.967** | **0.834** | **0.863** |
| Large inter-branch | **0.661** | **0.519** | **0.742** | - | 0.146 | 0.411 | - | 0.090 | - |
| Overhang by fine branching | - | 0.104 | 0.023 | - | 0.046 | - | - | 0.057 | 0.034 |
| Overhang by coarse strure | **0.239** | 0.016 | 0.127 | **0.181** | 0.420 | 0.070 | 0.033 | 0.019 | 0.104 |
| Uneven | - | - | - | - | - | - | - | - | - |
| Flat | - | - | - | - | - | - | - | - | - |
| Macroalge | - | - | - | - | - | - | - | - | - |
