# Supplementary material for: Nocturnal substrate association of four coral reef fish groups (parrotfishes, surgeonfishes, groupers and butterflyfishes) in relation to substrate architectural characteristics
Source: PeerJ. 2024 Jul 19;12:e17772. doi: 10.7717/peerj.17772 (PMC11262305; doi:10.7717/peerj.17772)
Supplement: Supplemental Information 18 — Significant positive associations are shown as bold characters. N.S.: non significant associations. -: no fishes were found on the substrates. [file peerj-12-17772-s018.docx]

| Substrate  architectural characteristics | Substrate type | *Chlorurus microrhinos* | *Chlorurus spilurus* | *Hipposcarus longiceps* | *Scarus ghobban* | *Scarus forsteni* | *Scarus niger* | *Scarus oviceps* | *Scarus rivulatus* | *Scarus schlegeli* |
| --- | --- | --- | --- | --- | --- | --- | --- | --- | --- | --- |
| Eave-like | Corymbose *Acropora* | 0.013 | 0.052 | 0.037 | 0.087 | 0.048 | 0.194 | 0.041 | 0.029 | **0.330** |
|  | Tabular *Acropora* | - | 0.085 | 0.046 | 0.030 | **0.471** | 0.790 | **0.174** | **0.600** | 0.105 |
|  | Foliose coral | - | - | - | - | - | - | 0.282 | - | 0.199 |
|  | Dead corymbose *Acropora* | 0.380 | 0.255 | - | - | - | - | - | - | - |
|  | Dead tabular *Acropora* | 0.228 | 0.306 | 0.326 | 0.218 | 0.421 | - | 0.355 | 0.253 | 0.251 |
|  | Dead foliose coral | - | - | - | - | - | - | - | - | - |
| Large | Staghorn *Acropora* | 0.264 | **0.155** | **0.470** | 0.031 | - | - | 0.128 | 0.037 | - |
| Inter-branch | Dead staghorn *Acropora* | - | - | - | - | - | - | - | - | - |
| Overhang by | Branching *Acropora* | - | - | - | - | - | - | - | - | - |
| fine branching | Bottlebrush *Acropora* | - | 0.011 | - | - | - | - | - | - | - |
|  | Non-acroporid branching coral | - | 0.079 | 0.048 | 0.032 | - | - | - | 0.074 | 0.037 |
|  | *Pocillopora* | - | - | - | - | - | - | - | - | - |
|  | Dead branching *Acropora* | - | - | - | - | - | - | - | - | - |
|  | Dead bottlebruch *Acropora* | - | - | - | - | - | - | - | - | - |
|  | Dead non-acroporid branching coral | - | 0.053 | - | - | - | - | - | - | - |
|  | Dead *Pocillopora* | - | - | - | - | - | - | - | - | - |
| Overhang by | Massive coral | 0.035 | - | - | 0.148 | - | - | - | - | 0.057 |
| coarse structure | Dead massive coral | - | - | - | 0.404 | - | - | - | - | - |
|  | Rock | 0.080 | 0.004 | 0.074 | 0.049 | 0.059 | 0.016 | 0.020 | 0.007 | 0.021 |
| Uneven | Other coral | - | - | - | - | - | - | - | - | - |
|  | Dead other coral | - | - | - | - | - | - | - | - | - |
|  | Soft coral | - | - | - | - | - | - | - | - | - |
| Flat | Coral rubble | - | - | - | - | - | - | - | - | - |
|  | Sand | - | - | - | - | - | - | - | - | - |
| Macroalgae | Macroalgae | - | - | - | - | - | - | - | - | - |
|  |  |  |  |  |  |  |  |  |  |  |
|  | |  | |  | |  |  |  |  | |
