# Supplementary material for: Nocturnal substrate association of four coral reef fish groups (parrotfishes, surgeonfishes, groupers and butterflyfishes) in relation to substrate architectural characteristics
Source: PeerJ. 2024 Jul 19;12:e17772. doi: 10.7717/peerj.17772 (PMC11262305; doi:10.7717/peerj.17772)
Supplement: Supplemental Information 19 — Significant positive associations are shown as bold characters. N.S.: non significant associations. -: no fishes were found on the substrates. [file peerj-12-17772-s019.docx]

| Substrate  architectural characteristics | *Naso unicornis* | *Naso lituratus* |  | *Plectropomus leopardus* | *Epinephelus ongus* |  | *Chaetodon trifascialis* | *Chaetodon lunulatus* | *Chaetodon ephippium* | *Chaetodon auriga* |
| --- | --- | --- | --- | --- | --- | --- | --- | --- | --- | --- |
| Eave-like | 0.292 | **0.651** |  | **0.494** | 0.172 |  | **0.307** | 0.059 | - | 0.371 |
| Large inter-branch | - | - |  | 0.169 | 0.378 |  | **0.633** | **0.871** | **0.824** | 0.305 |
| Overhang by fine branching | 0.038 | 0.114 |  | 0.133 | 0.222 |  | 0.044 | 0.057 | - | 0.096 |
| Overhang by coarse structure | **0.670** | 0.235 |  | 0.181 | **0.228** |  | 0.015 | 0.013 | 0.176 | 0.229 |
| Uneven | - | - |  | - | - |  | - | - | - | - |
| Flat | - | - |  | 0.023 | - |  | - | - | - | - |
| Macroalge | - | - |  | - | - |  | - | - | - | - |
|  |  |  |  |  |  |  |  |  |  |  |
