# Supplementary material for: Nocturnal substrate association of four coral reef fish groups (parrotfishes, surgeonfishes, groupers and butterflyfishes) in relation to substrate architectural characteristics
Source: PeerJ. 2024 Jul 19;12:e17772. doi: 10.7717/peerj.17772 (PMC11262305; doi:10.7717/peerj.17772)
Supplement: Supplemental Information 20 — Significant positive associations are shown as bold characters. N.S.: non significant associations. -: no fishes were found on the substrates. [file peerj-12-17772-s020.docx]

| Substrate  architectural characteristics | Substrate type | *Naso unicornis* | *Naso lituratus* |  | *Plectropomus leopardus* | *Epinephelus ongus* |  | *Chaetodon trifascialis* | *Chaetodon lunulatus* | *Chaetodon ephippium* | *Chaetodon auriga* |
| --- | --- | --- | --- | --- | --- | --- | --- | --- | --- | --- | --- |
| Eave-like | Corymbose *Acropora* | 0.000 | 0.033 |  | 0.053 | 0.034 |  | 0.000 | 0.039 | - | 0.107 |
|  | Tabular *Acropora* | 0.131 | 0.108 |  | 0.098 | 0.058 |  | **0.447** | 0.048 | - | - |
|  | Foliose coral | - | - |  | - | - |  | - | - | - | - |
|  | Dead corymbose *Acropora* | - | - |  | 0.195 | - |  | - | - | - | - |
|  | Dead tabular *Acropora* | 0.234 | - |  | 0.117 | 0.177 |  | - | - | - | 0.622 |
|  | Dead foliose coral | - | - |  | - | - |  | - | - | - | - |
| Large | Staghorn *Acropora* | - | - |  | 0.034 | 0.128 |  | **0.462** | **0.705** | 0.160 | 0.090 |
| inter-branch | Dead staghorn *Acropora* | - | - |  | - | 0.027 |  | - | - | - | - |
| Overhang by | Branching *Acropora* | - | - |  | - | 0.013 |  | 0.081 | 0.078 | - | 0.035 |
| fine branching | Bottlebrush *Acropora* | - | - |  | - | 0.017 |  | - | 0.017 | - | - |
|  | Non-acroporid branching coral | 0.034 | 0.014 |  | 0.051 | **0.173** |  | - | 0.017 | - | 0.046 |
|  | *Pocillopora* | - | 0.076 |  | 0.091 | 0.092 |  | - | 0.088 | - | - |
|  | Dead branching *Acropora* | - | - |  | - | 0.041 |  | - | - | - | - |
|  | Dead bottlebruch *Acropora* | - | - |  | - | - |  | - | - | - | - |
|  | Dead non-acroporid branching coral | - | - |  | 0.081 | 0.123 |  | - | - | - | - |
|  | Dead *Pocillopora* | - | 0.720 |  | - | - |  | - | - | - | - |
| Overhang by | Massive coral | - | 0.029 |  | 0.035 | 0.053 |  | - | - | - | 0.047 |
| coarse structure | Dead massive coral | 0.435 | - |  | 0.217 | - |  | - | - | 0.822 | - |
|  | Rock | **0.165** | 0.019 |  | 0.023 | 0.065 |  | 0.010 | 0.010 | 0.019 | 0.053 |
| Uneven | Other coral | - | - |  | - | - |  | - | - | - | - |
|  | Dead other coral | - | - |  | - | - |  | - | - | - | - |
|  | Soft coral | - | - |  | - | - |  | - | - | - | - |
| Flat | Coral rubble | - | - |  | 0.004 | - |  | - | - | - | - |
|  | Sand | - | - |  | - | - |  | - | - | - | - |
| Macroalge | Macroalgae | - | - |  | - | - |  | - | - | - | - |
