# Supplementary material for: Nocturnal substrate association of four coral reef fish groups (parrotfishes, surgeonfishes, groupers and butterflyfishes) in relation to substrate architectural characteristics
Source: PeerJ. 2024 Jul 19;12:e17772. doi: 10.7717/peerj.17772 (PMC11262305; doi:10.7717/peerj.17772)
Supplement: Supplemental Information 22 — Significant positive associations are shown as bold characters. N.S.: non significant associations. -: no fishes were found on the substrates. [file peerj-12-17772-s022.docx]

| Substrate  architectural characteristics | Substrate type | Parrotfishes (Labridae : Scarini) |  | Surgeonfishes (Acanthuridae) |  | Groupers (Epinephelidae) |  | Butterflyfishes (Chaetodontidae) |
| --- | --- | --- | --- | --- | --- | --- | --- | --- |
| Eave-like | Corymbose *Acropora* | **Positive** |  | N.S. |  | N.S. |  | N.S. |
|  | Tabular *Acropora* | **Positive** |  | **Positive** |  | N.S. |  | N.S. |
|  | Foliose coral | N.S. |  | - |  | - |  | - |
|  | Dead corymbose *Acropora* | N.S. |  | - |  | N.S. |  | - |
|  | Dead tabular *Acropora* | **Positive** |  | N.S. |  | N.S. |  | N.S. |
|  | Dead foliose coral | - |  | - |  | - |  | - |
| Large inter-branch | Staghorn *Acropora* | **Positive** |  | - |  | N.S. |  | **Positive** |
|  | Dead staghorn *Acropora* | - |  | - |  | N.S. |  | - |
| Overhang by fine branching | Branching *Acropora* | - |  | - |  | Negative |  | N.S. |
|  | Bottlebrush *Acropora* | Negative |  | - |  | Negative |  | Negative |
|  | Non-acroporid branching coral | N.S. |  | N.S. |  | **Positive** |  | N.S. |
|  | *Pocillopora* | - |  | N.S. |  | N.S. |  | N.S. |
|  | Dead branching *Acropora* | - |  | - |  | N.S. |  | - |
|  | Dead bottlebruch *Acropora* | - |  | - |  | - |  | - |
|  | Dead non-acroporid branching coral | N.S. |  | - |  | N.S. |  | - |
|  | Dead *Pocillopora* | - |  | N.S. |  | - |  | - |
| Overhang by coarse structure | Massive coral | N.S. |  | N.S. |  | N.S. |  | Negative |
|  | Dead massive coral | N.S. |  | N.S. |  | N.S. |  | N.S. |
|  | Rock | N.S. |  | **Positive** |  | N.S. |  | N.S. |
| Uneven | Other coral | - |  | - |  | - |  | - |
|  | Dead other coral | - |  | - |  | - |  | - |
|  | Soft coral | - |  | - |  | - |  | - |
| Flat | Coral rubble | - |  | - |  | Negative |  | - |
|  | Sand | - |  | - |  | - |  | - |
| Macroalge | Macroalgae | - |  | - |  | - |  | - |
|  |  |  |  |  |  |  |  |  |
