# Supplementary material for: Nocturnal substrate association of four coral reef fish groups (parrotfishes, surgeonfishes, groupers and butterflyfishes) in relation to substrate architectural characteristics
Source: PeerJ. 2024 Jul 19;12:e17772. doi: 10.7717/peerj.17772 (PMC11262305; doi:10.7717/peerj.17772)
Supplement: Supplemental Information 23 — Significant positive associations are shown as bold characters. N.S.: non significant associations. -: no fishes were found on the substrates. [file peerj-12-17772-s023.docx]

| Substrate  architectural characteristics | Parrotfishes (Labridae : Scarini) |  | Surgeonfishes (Acanthuridae) |  | Groupers (Epinephelidae) |  | Butterflyfishes (Chaetodontidae) |
| --- | --- | --- | --- | --- | --- | --- | --- |
| Eave-like | **0.129** |  | **0.471** |  | 0.246 |  | 0.129 |
| Large inter-branch | **0.731** |  | 0.000 |  | 0.338 |  | **0.731** |
| Overhang by fine branching | 0.058 |  | 0.076 |  | 0.190 |  | 0.058 |
| Overhang by coarse structure | 0.082 |  | **0.454** |  | **0.221** |  | 0.082 |
| Uneven | - |  | - |  | - |  | - |
| Flat | - |  | - |  | 0.005 |  | - |
| Macroalge | - |  | - |  | - |  | - |
|  |  |  |  |  |  |  |  |
