# Supplementary material for: Nocturnal substrate association of four coral reef fish groups (parrotfishes, surgeonfishes, groupers and butterflyfishes) in relation to substrate architectural characteristics
Source: PeerJ. 2024 Jul 19;12:e17772. doi: 10.7717/peerj.17772 (PMC11262305; doi:10.7717/peerj.17772)
Supplement: Supplemental Information 24 — Significant positive associations are shown as bold characters. N.S.: non significant associations. -: no fishes were found on the substrates. [file peerj-12-17772-s024.docx]

| Substrate  architectural characteristics | Substrate type | Parrotfishes (Labridae : Scarini) |  | Surgeonfishes (Acanthuridae) |  | Groupers (Epinephelidae) |  | Butterflyfishes (Chaetodontidae) |
| --- | --- | --- | --- | --- | --- | --- | --- | --- |
| Eave-like | Corymbose *Acropora* | **0.075** |  | 0.024 |  | 0.045 |  | 0.042 |
|  | Tabular *Acropora* | **0.170** |  | **0.115** |  | 0.065 |  | 0.083 |
|  | Foliose coral | 0.033 |  | - |  | - |  | - |
|  | Dead corymbose *Acropora* | 0.138 |  | - |  | 0.060 |  | - |
|  | Dead tabular *Acropora* | **0.311** |  | 0.069 |  | 0.180 |  | 0.091 |
|  | Dead foliose coral | - |  | - |  | - |  | - |
| Large inter-branch | Staghorn *Acropora* | **0.124** |  | - |  | 0.094 |  | **0.414** |
|  | Dead staghorn *Acropora* | - |  | - |  | 0.033 |  | - |
| Overhang by fine branching | Branching *Acropora* | - |  | - |  | 0.012 |  | 0.052 |
|  | Bottlebrush *Acropora* | 0.006 |  | - |  | 0.011 |  | 0.007 |
|  | Non-acroporid branching coral | 0.040 |  | 0.020 |  | **0.122** |  | 0.020 |
|  | *Pocillopora* | - |  | 0.053 |  | 0.084 |  | 0.071 |
|  | Dead branching *Acropora* | - |  | - |  | 0.025 |  | - |
|  | Dead bottlebruch *Acropora* | - |  | - |  | - |  | - |
|  | Dead non-acroporid branching coral | 0.014 |  | - |  | 0.100 |  | - |
|  | Dead *Pocillopora* | - |  | 0.509 |  | - |  | - |
| Overhang by coarse structure | Massive coral | 0.022 |  | 0.021 |  | 0.049 |  | 0.014 |
|  | Dead massive coral | 0.039 |  | 0.128 |  | 0.067 |  | 0.169 |
|  | Rock | 0.028 |  | **0.062** |  | 0.052 |  | 0.037 |
| Uneven | Other coral | - |  | - |  | - |  | - |
|  | Dead other coral | - |  | - |  | - |  | - |
|  | Soft coral | - |  | - |  | - |  | - |
| Flat | Coral rubble | - |  | - |  | 0.001 |  | - |
|  | Sand | - |  | - |  | - |  | - |
| Macroalge | Macroalgae | - |  | - |  | - |  | - |
|  |  |  |  |  |  |  |  |  |
